# Role of the clock gene homolog *aha-1* in the circadian system of *Caenorhabditis elegans*

**DOI:** 10.3389/fnins.2025.1618370

**Published:** 2025-06-24

**Authors:** Melisa Luciana Lamberti, Francisco Silva, María Eugenia Goya, Claire Y. Bénard, Diego A. Golombek, María Laura Migliori

**Affiliations:** ^1^Laboratorio de Cronobiología, Departamento de Ciencia y Tecnología, Universidad Nacional de Quilmes, Bernal, Argentina; ^2^ERIBA-European Research Institute for the Biology of Ageing, Groningen, Netherlands; ^3^Département des Sciences Biologiques, Université du Québec a Montréal, Montreal, QC, Canada; ^4^CERMO-FC Research Center, Montreal, QC, Canada; ^5^Laboratorio Interdisciplinario del Tiempo (LITERA), Universidad de San Andrés/CONICET, Victoria, Argentina

**Keywords:** *C. elegans*, circadian rhythms, luminescence, clock gene, light, temperature

## Abstract

**Background:**

Circadian rhythms are endogenous and allow organisms to adapt to external daily rhythms of light, temperature and other environmental factors. Circadian rhythms are regulated by a central clock, which is based on a transcription-translation feedback loop. The AHA-1 protein from *Caenorhabditis elegans* possesses all conserved domains and shows high homology with the positive elements of the central clock loop, BMAL1 in mammals and CYCLE in *Drosophila*.

**Methods:**

We studied the possible involvement of *aha-1* in the circadian system of adult *C. elegans* using a bioluminescence-based circadian transcriptional reporter. We also performed qPCRs experiments to quantify the mRNA levels for the *aha-1* gene from bulk RNA extractions from adult worms.

**Results:**

We observed robust luminescent circadian rhythms driven by the *aha-1* promoter. However, *aha-1* mRNA levels did not show circadian oscillation under the conditions tested. We also show that a mutation in *aha-1* generates a significantly longer endogenous period than the one in control strains, suggesting a role for this gene in the nematode circadian clock.

**Conclusion:**

The results indicate that the CYCLE/BMAL-1 homolog AHA-1 plays a key role in the generation of circadian rhythms in adult nematodes, mainly by regulating period length. These results suggest that the molecular control of circadian regulation in *C. elegans* exhibits some similarities to other clock model systems.

## 1 Introduction

Circadian rhythms have a period close to 24 h, are maintained under constant conditions, and are synchronized by environment signals (“time givers”, or zeitgebers) in order to adapt to external daily changes of light, temperature, and other environmental factors. For most organisms the main environmental synchronizer is the light–dark cycle, but stimuli such as temperature are also capable of synchronizing the clock (George and Stanewsky, 2021). The molecular machinery of circadian clocks consists of transcriptional-translational feedback loops (TTFL) that maintain cycles of gene expression with a period of approximately 24 h (King and Sehgal, 2020; Patke et al., 2020; Cox and Takahashi, 2019). In mammals, BMAL1 and CLOCK proteins form a heterodimer that acts as a positive element in the core feedback loop, activating the transcription of PER (PERIOD) and CRY (CRYPTOCROME) and many other clock-controlled genes, by binding to the E-boxes in their promoters. In addition, PER: CRY: CK1ε/*δ* heterodimers function as the negative element in the feedback loop by inhibiting the activity of CLOCK: BMAL1 (Cox and Takahashi, 2019; Spangler et al., 2025). On the other hand, CK1ε/*δ* protein acts by tagging PER monomers for degradation, resulting in daily changes in PER levels and CLOCK/BMAL1 activity (Eide et al., 2005). In *D. melanogaster*, dmCLOCK and dmCYCLE make up the positive elements while dmPER and dmTIMELESS function as the negative elements of the loop (Rosato et al., 2006; King and Sehgal, 2020). In flies, CRY acts mainly as a photoreceptor, directly sensing light and passing the information to the core loop via control of TIM stability (Spangler et al., 2025). On the other hand, the CK1ε homolog in flies, DOUBLETIME, induces PER degradation through phosphorylation (Claridge-Chang et al., 2001).

Different circadian rhythms have been described in *C. elegans* (Migliori et al., 2023); furthermore, some molecular components of its central pacemaker are currently known (Spangler et al., 2025; Simonetta et al., 2009; Romanowski et al., 2014; Lamberti et al., 2024). *C. elegans*’ AHA-1 protein shows different degrees of homologies with BMAL1 from mammals and CYCLE from *Drosophila* (Romanowski et al., 2014). AHA-1 is involved in several processes, including cell differentiation, determination of adult lifespan, and social behavior (Huang et al., 2004; Jacobi et al., 2015); however, its function in circadian rhythms has not been studied at the molecular level. Genome-wide expression has revealed no significant circadian oscillations in *aha-1* mRNA levels (van der Linden et al., 2010), at least under classic light/dark or temperature cycles and in total RNA extracts from whole animals.

The objective of this work is to explore the role of the clock gene homolog *aha-1* in the circadian system of the adult nematode using a bioluminescence-based circadian transcriptional reporter.

## 2 Materials and methods

### 2.1 Nematodes strains

*C. elegans* strains N2 (Bristol strain, wild-type) was provided by the Caenorhabditis Genetics Center (CGC), University of Minnesota, MN, United States. Strain XD321 *aha-1(xd4)* I was provided by the Mei Ding laboratory. This strain has a G/A substitution at the second splicing site, resulting in a decrease in the expression levels of *aha-1* (Zhang et al., 2013). Strains VQ1310 *qvIs8* [*psur-5::luc::gfp + punc-122::RFP*], VQ1071 *qvEx295* [*paha-1::luc::gfp*] and VQ1324 *qvEx361* [*plin-42::luc::gfp::pest, pCFJ90*] were previously generated in the laboratory. The XD321 strain was crossed with the VQ1310 *qvIs8* strain, generating the VQ1722 *aha-1(xd4)* I*; qvIs8* [*psur-5::luc::gfp + punc-122::RFP*] strain.

Transgenic nematodes were generated by standard microinjection techniques (Mello and Fire, 1995) through a collaboration with the laboratory of Dr. Claire Bénard (Université du Québec à Montréal, Canada). The integration of *psur-5::luc::gfp* was induced by UV radiation to generate *qvIs8* (Mariol et al., 2013).

### 2.2 Growth conditions

*C. elegans* strains were maintained on NGM medium (0.3% NaCl, 0.25% Peptone, 5 μg/mL cholesterol, 1 mmoL/L CaCl_2_, mmol/l MgSO_4_, 1.7% Agar in 25 mmoL/L of potassium phosphate buffer pH 6.0) with thick bacterial lawns of *Escherichia coli* HB101 strain under a dual cycle of light and temperature, Light/Dark (LD, ~150/0 μmol/m^2^/s) and Cold/Warm (CW, 18.5/20°C, *Δ* = 1.5°C ± 0.5°C) cycles 12:12 h. Light and temperature are known to be the main zeitgebers, and in this entrainment protocol ZT0 (9:00 AM) indicates the time of lights on and the cold phase onset. Circadian Time (CT) refers to a specific time in the free-running (FR) conditions (i.e., DD/WW cycles). Photo and thermal conditions were controlled with an I-291PF incubator (INGELAB, Argentina) and temperature was monitored using DS1921H-F5 iButton Thermochrons (Maxim Integrated, United States). Four cool white LED light strips (approx.150 μmol/m^2^/s) were used as the light source for the luminescence recordings. Nematode populations were synchronized to the same developmental stage by the chlorine method (Stiernagle, 2006).

### 2.3 Luminescence assays

Luminescence recording was carried out according to a protocol developed by the laboratory (Goya et al., 2016). Briefly, approximately 50 L4 hermaphrodite nematodes were manually selected for their higher GFP expression by picked under an SMZ100 stereomicroscope equipped with an epi-fluorescence attachment (Nikon) with a cool Multi-TK-LED light source (Tolket) to avoid warming of the plate. The nematode populations were washed once with M9 buffer (42 mM Na_2_HPO_4_, 22 mM KH_2_PO_4_, 85.5 mM NaCl, 1 mM MgSO_4_) to remove all traces of bacteria and resuspended in 200 μL of luminescence medium: Leibovitz’s L-15 media without phenol red (Thermo Fisher Scientific) supplemented with 1X antibiotic–antimycotic (Thermo Fisher Scientific), 40 μM of 5-fluoro-2′-deoxyuridine (FUdR) to avoid new eclosions, 5 mg/mL of cholesterol, 10 μg/mL of tobramycin (Tobrabiotic, Denver Farma), 1 mM of D-luciferin (Gold Biotechnology) and 0.05% Triton X-100 to increase cuticle permeabilization. Nematode populations were then transferred to a white, flat-bottomed 96-well plate (Greiner), sealed with optical film (Microseal B PCR Plate Sealing Film, Biorad) and perforated to prevent condensation and allow oxygen exchange. Then, at ZT12 (21 h) of the same day, the plate was placed in a Berthold Centro LB 960 microplate luminometer (Berthold Technologies) stationed inside an incubator model G291PF (INGELAB, Argentina) to allow tight control of the light and temperature in each experiment. Microwin 2000 software 4.43 (Mikrotek 2 Laborsysteme) was programmed to leave the plate outside the luminometer after each recording to expose nematodes to the environmental cues. The luminescence of each well was integrated for 10 s every 30 min. Luminescence was monitored for 3 days under a dual 24 h LD/CW (CW, 15.5/17°C, *Δ* = 1.5 ± 0.5°C) cycle and then 3 days under FR (DD/WW; 17°C) conditions. The signal emitted by the nematodes was integrated for 10 s and recorded with an interval of 30 min.

### 2.4 Data acquisition and analysis

Luminescence was sampled at 30 min intervals. Background noise was extracted from the raw data obtained from the luminometer. In all cases, the first 12 to 24 h of recording were removed due to accumulation of the luciferase enzyme. All raw data were analyzed using a Shiny app developed in the laboratory.^1^ The raw data were detrended, smoothed and normalized to the initial maximum value of each sample and plotted using R. All data are shown as mean ± SEM of luminescence as indicated in the figures. In each case, the mean corresponds to a population of nematodes (50 nematodes). Subsequently, the period under LD/CW cycles and FR conditions was calculated from the population data using the Lomb-Scargle (LS) periodogram using the lomb R package (Van Dongen et al., 1999), within a range of 18 to 37 h and with an oversampling of 30. The acrophase (time at peak) and amplitude of each signal was estimated using the Cosinor method, by fitting a cosine waveform to the data using a non-linear least squares regression implemented using the nls function of base R and obtaining the R^2^ of the fit. Any signal resulting from population analysis with a 24 h period and an R^2^ adjustment ≥ 0.5 was considered “Synchronized” under cyclic training conditions. In the case of FR rhythms, any signal resulting from the analysis with a period range between 18 h and 37 h, and an R^2^ adjustment ≥ 0.5 was considered “Circadian.” For statistical analyses, the GraphPad Prism 7 software was used. Statistical significance was set at alpha = 0.05. Final figures were generated using Biorender.^2^

### 2.5 Quantitative real-time PCR

Nematode populations (N2 strain) were synchronized to the same developmental stage by the chloride method (Stiernagle, 2006) and cultured overnight in a 50 mL Erlenmeyer flask with 3.5 mL of M9 buffe, 1X antibiotic-antimycotic (Thermo Fisher Scientific) and 10 μg/mL of tobramycin (Tobrabiotic, Denver Farma) at 110 rpm in LD/CW cycle (18.5/20°C). The next day, L1 larvae were placed on NGM plate and were grown for 48 h to the L4 stage under the same LD/CW cycle. Then, independent populations of approximately 4,000 nematodes at the L4 stage were washed with M9 buffer to remove the remains of bacteria and transferred to four 500-ml flasks with 130 mL of luminescence medium without D-luciferin (5 nematodes/10 μL). The nematodes then were cultured under LD/CW (15.5/17°C) cycle with agitation at 100 rpm for two additional days. Then they were released in FR conditions (DD/WW; 17°C) for the two remaining days of the assay. Four independent biological replicates (*n* = 4; 4,000 adult nematodes in each replicate) were collected every 4 h, starting at ZT1 (10 AM) in the LD/CW (15.5/17°C) cycle during the last day of entrainment and during the following 2 days of the FR condition (DD/WW; 17°C). Nematodes were centrifuged to discard the medium, washed, and frozen at −80°C in Trizol (Thermo Fisher Scientific). Total RNA was extracted from the samples using the Trizol method according to the manufacturer’s instructions. RNA solutions were quantified using NanoDrop1000 (Thermo Fisher Scientific), and their integrity was evaluated by electrophoresis. Two micrograms of total RNA and poly-T primers (20 nt long) (Thermo Fisher Scientific) were used for cDNA synthesis using SuperScript II Reverse Transcriptase (Thermo Fisher Scientific). Gene amplification was performed on a QuantStudio 3 PCR instrument (Thermo Fisher Scientific), using 10 μL of final reaction volume containing 1 μL of cDNA as the template, 1 × of the SYBR Green PCR Master Mix 3.0 (PB-L), and the corresponding primers at a final concentration of 0.2–0.6 μM. The cDNA template was amplified in duplicate, with the following conditions: 95°C for 10 min, followed by 45 cycles of 95°C for 15 s and 60°C for 1 min. Relative gene expression was calculated using the 2r^− ΔΔCt^ method, and Y45F10D.4 was the reference housekeeping gene. For pPCR analyses, the JTK_CYCLE algorithm implemented in the *MetaCycle* package (R) was used (*p* = 1; not statistically significant).

The primers used for amplification were:

Fw-aha-1: 5´-GTTCGTGTTTCGGAAGATGG-3′,

Rv-aha-1: 5´-TTCTCATCTGCTGGATGTGC-3′,

Fw-Y45f10D.4: 5´-GTCGCTTCAAATCAGTTCAGC-3′,

Rv-Y45f10D.4: 5´-GTTCTTGTCAAGTGATCCGACA-3′.

## 3 Results

### 3.1 *aha-1* gene expression exhibits circadian rhythms

**Figure 1:**
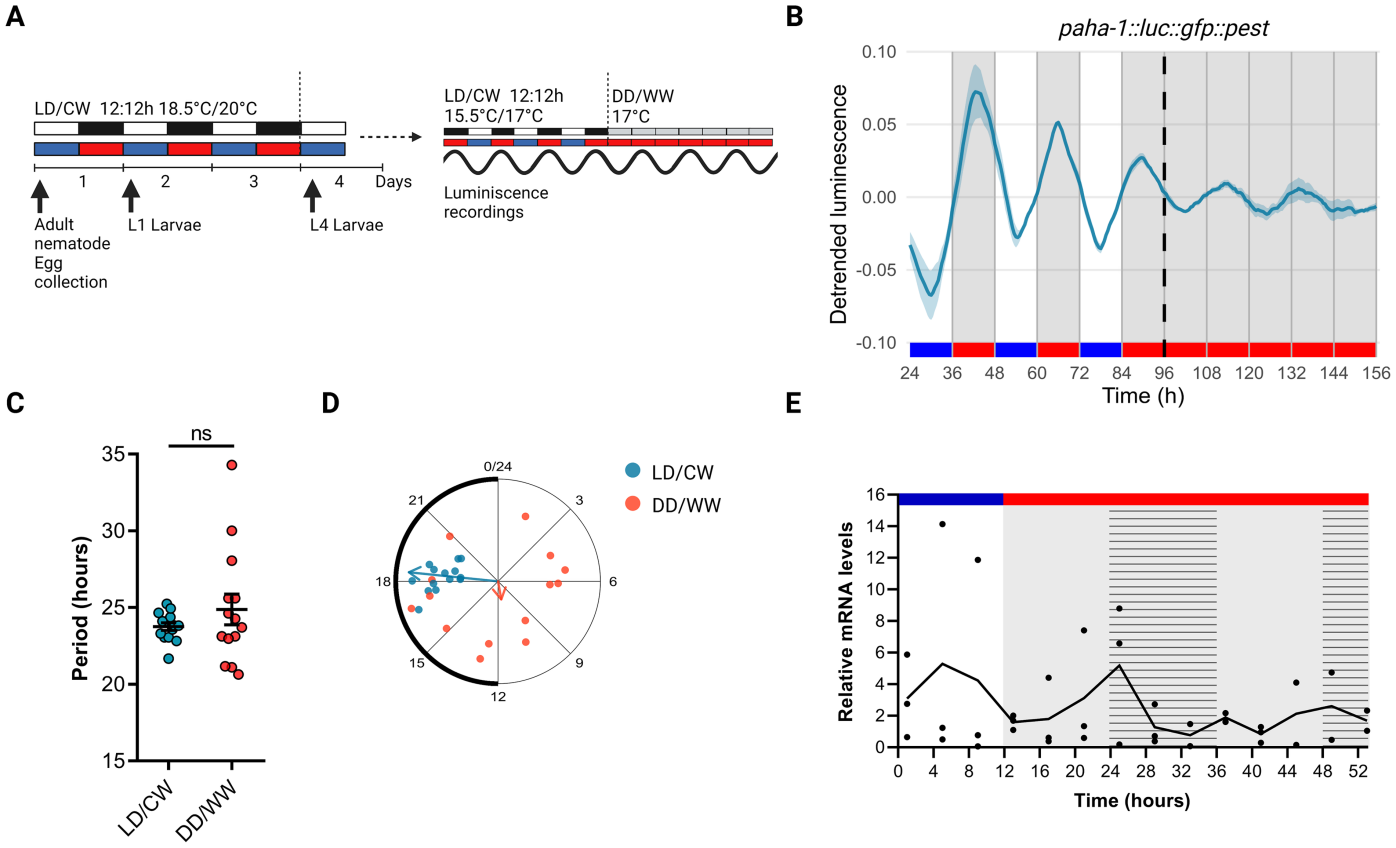
Circadian rhythms in the expression of the *aha-1* gene. **(A)** Schematic assay of the luminescence experiments and photo/thermoperiodic conditions. Black/white bars indicate dark/light; blue/red bars indicate cold/warm; grey and red bars indicate the FR conditions. VQ1071 *qvEx295* [*aha-1::luc::gfp::pest*] strain were grown under dual cyclic conditions of 12:12 h Light/Dark (LD, ~ 150/0 μmol/m^2^/s) and cold/warm (CW, 18.5/20°C); ZT0, lights on and cold temperature phase onset, on NGM plates with a lawn of *E. coli* HB101. Embryos were collected from adult nematodes, hatched in the absence of food, and staged L1 (larval stage 1) nematodes were grown on NGM plates with bacteria for three days. Once at the L4 stage, approximately 50 nematodes were transferred to the liquid luminescence media. Luminescence assays were performed under dual cyclic conditions 12:12 h of LD (~ 150/0 μmol/m2/s) and CW (15.5/17°C) for three days; ZT0, lights on and cold temperature phase onset. Then, the luminescence was measured under FR conditions (DD/WW, 17°C). **(B)** Average reporter activity of adult populations under dual cyclic conditions (LD/CW, 15.5/17°C) and FR conditions (DD/WW, 17°C). Luminescence signals are shown as mean ± SEM. The average reported activity was displayed with rhythmic populations (*n* = 14). Each population consisted of 50 adult nematodes per well. **(C)** Average period of rhythmic populations (LD/CW: 23.76 ± 0.25 h; DD/WW: 24.88 ± 1.00 h; *n* = 14). Paired t-test, *p* = 0.24; normality of paired differences was confirmed (Shapiro–Wilk, *p* = 0.394). **(D)** Rayleigh plots showing the phase of the bioluminescent peak under dual cyclic conditions (LD/CW, light blue dots) and the first bioluminescent peak on the first day of release to FR (DD/WW, pink dots) for rhythmic population nematodes (LD/CW: 18.38 ± 0.23 h, *n* = 14; R = 0.98 and DD/WW: 11.33 ± 1.79 h, *n* = 14; R = 0.1). **(E)** *aha-1* expression levels determined by qPCR from bulk RNA extractions from adult worms. Four independent biological replicates (*n* = 4; 4,000 adult nematodes in each replicate) were collected every 4 h, starting at ZT1 (10 AM) in the LD/CW (15.5/17°C) cycle during the last day of entrainment and during the following 2 days of the FR condition (DD/WW; 17°C). Striped bar indicate subjective day and subjective cold temperature. JTK_CYCLE analysis (MetaCycle, R) on qPCR data: *p* = 1 (not significant).

To assess a possible circadian expression in the expression of the *aha-1* gene, we recorded luminescence rhythms from VQ1071 *qvEx295* [*paha-1::luc::gfp::pest*] strain. Animals were entrained to a dual LD/CW cycle (18.5/20°C, *Δ* = 1.5°C) and the bioluminescent activity of the *aha-1* promoter was measured thereafter in adult nematodes over three days in LD/CW (15.5/17°C, Δ = 1.5°C) cycles and then for three days under constant conditions (DD/WW, 17°C) (Figure 1A). We choose the 15.5/17°C cycle for recordings because 15.5°C is the minimal temperature allowed by the luminometer used and because the populations have a longer life span under this condition than at 18.5/20°C, resulting in a sustained luminescence signal for more days than at higher temperatures. Importantly, the temperature delta (1.5°C) is the same in both conditions. The VQ1071 strain exhibited a rhythm in the activity of the *aha-1* promoter under cyclical and FR conditions, which increased during the nocturnal/warm phase and decreased during the cold phase (Figure 1B; Supplementary Figure S1). Under LD/CW and FR conditions, rhythmic adult populations showed a period of 23.76 ± 0.25 h and 24.88 ± 1.00 h, respectively (*n* = 14 mutant rhythmic, *n* total = 30, ~ 47% rhythmic) (Figure 1C; Supplementary Table S1). Approximately 32% of the rhythmic populations were synchronized following this entrainment protocol, as indicated by the acrophase distribution under cyclic conditions (LD/CW, light blue dots) and FR (DD/WW, pink dots) (Figure 1D). Supplementary Table S2 shows circadian period estimation by different methods, showing similar results.

To gain insights into the *aha-1* expression pattern, we performed qPCRs experiments to quantify the mRNA levels for the *aha-1* gene from bulk RNA extractions from adult worms. Samples were taken every 4 h for one day under cyclic conditions (LD/CW, 15.5/17°C) and for one day under constant conditions (DD/WW, 17°C), starting from the young adult stage. Although no circadian pattern for *aha-1* gene was revealed (JTK_CYCLE, *p* = 1), we did detect the expression of gene throughout the experiment (Figure 1E). A daily variation was observed under LD/CW conditions, with peak expression during the diurnal phase; the expression levels of *aha-1* decreased under constant conditions. It is still possible that orthologous clock genes do cycle in a small subset of cells or tissues in worms and that this get lost from bulk RNA extraction. Using published data single-cell RNA sequencing by CeNGEN (Hammarlund et al., 2018), which provides gene expression profiles of all *C. elegans* neurons in L4 nematodes, we found that the gene *aha-1* is expressed in sensory neurons, interneurons, motor neurons, and pharyngeal neurons (Supplementary Figure S2A). Furthermore, using scRNA-sequencing data from a published database of gene expression in young adult and aging *C. elegans* (Ghaddar et al., 2023; Roux et al., 2023), we see that, the gene *aha-1* is expressed in neurons and other cell types, in young adults and aging nematodes (Supplementary Figure S2B). Moreover, we examined the expression of *aha-1* across different adult stages using the publicly available RNA-seq dataset from the *C. elegans* Aging Atlas.^3^ According to this dataset, the expression levels of *aha-1* remain stable from day 1 to day 14 of adulthood in neuron as well as in other tissues (Supplementary Figure S2C). This suggests that the overall abundance of *aha-1* transcripts does not change significantly during aging.

**Figure 2:**
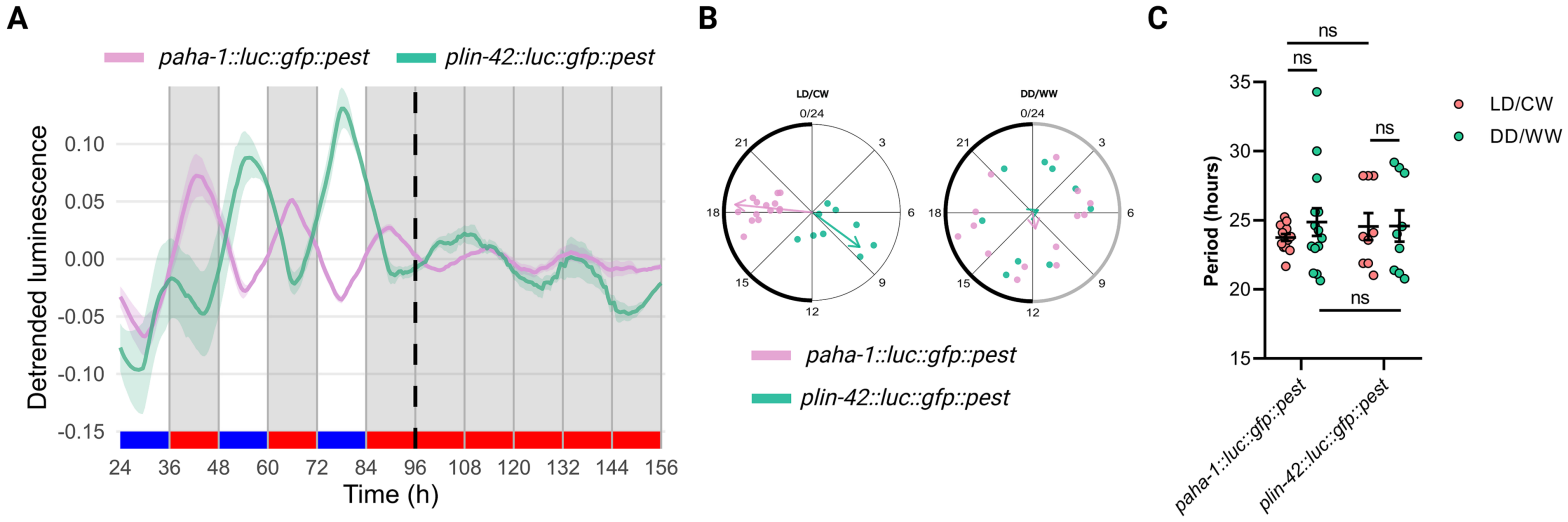
The luminescence rhythms for the *lin-42* promoter are in antiphase to the bioluminescence rhythm of the *aha-1* promoter under cyclic conditions. **(A)** Average reporter activity of adult populations under dual cyclic conditions (LD/CW, 15.5/17°C) and FR conditions (DD/WW, 17°C): *paha-1::luc::gfp::pest* and *plin-42::luc::gfp::pest* mutants. Luminescence signals are shown as mean ± SEM. The average reported activity was displayed with rhythmic populations. Each population consisted of 50 adult nematodes per well. **(B)** Average period of rhythmic populations for *paha-1::luc::gfp::pest* (LD/CW: 23.76 ± 0.25 h; DD/WW: 24.88 ± 1.00 h; *n* = 14) and *plin-42::luc::gfp::pest* (LD7CW: 24.55 ± 0.97 h; DD/WW: 24.59 ± 1.14 h; *n* = 9) mutant. Normality of paired differences was confirmed for both strains (Supplementary Table S1). Paired (LD/CW vs. DD/WW within strains) and Unpaired t-tests (between strains) showed no significant differences. **(C)** Rayleigh plots showing the phase of the bioluminescent peak under dual cyclic conditions (LD/CW) and the first bioluminescent peak on the first day of release to FR (DD/WW) for rhythmic population nematodes: *paha-1::luc::gfp::pest* (LD/CW: 18.38 ± 0.23 h, R = 0.98 and DD/WW: 11.33 ± 1.79 h, R = 0.10; *n* = 14, pink dots) and *plin-42::luc::gfp::pest* (LD/CW: 8.39 ± 0.74 h, R = 0.76 and DD/WW: 4.30 ± 2.27 h, R = 0.07; *n* = 9, green dots).

In *C. elegans*, *per* is conserved as *lin-42* (Romanowski et al., 2014). Our laboratory previously showed that mutations of *lin-42* generate a significantly longer endogenous period, suggesting a role for this gene in the nematode circadian clock, as in other organisms (Lamberti et al., 2024). We also examined luminescence rhythms of VQ1324 *qvEx361* [*aha-1::luc::gfp::pest*, *pCFJ90*] strain under cyclic conditions (LD/CW, 15.5/17°C) followed by constant dark and temperature conditions (DD/WW, dark and 17°C). Interestingly, the luminescence rhythms for the *lin-42* promoter were in antiphase to the bioluminescence rhythm of the *aha-1* promoter under cyclic conditions (Figures 2A,B). Under FR conditions, rhythmic adult populations showed a period of 24.59 ± 1.14 h (VQ1324, *n* = 9 rhythmic, *n* total = 26, 34.6% rhythmic) (Figure 2C; Supplementary Table S1). We also used publicly available RNA-seq data from the *C. elegans* Aging Atlas (see text footnote 3) (Gao et al., 2024), and found that *aha-1*, *lin-42*, and *kin-20* (another critical regulator of the adult nematode circadian clock through neuronal cells) are expressed in pharyngeal and sensory neurons (Supplementary Figure S2D). This supports the hypothesis of a potential interaction between *aha-1* and *lin-*42. The specific cells involved in the circadian clock of *C. elegans* remain unidentified, but neurons are likely candidates, as observed in other organisms. Silencing candidate genes in neurons will be necessary to assess their potential role.

### 3.2 *aha-1* modulates period length in adult nematodes

**Figure 3:**
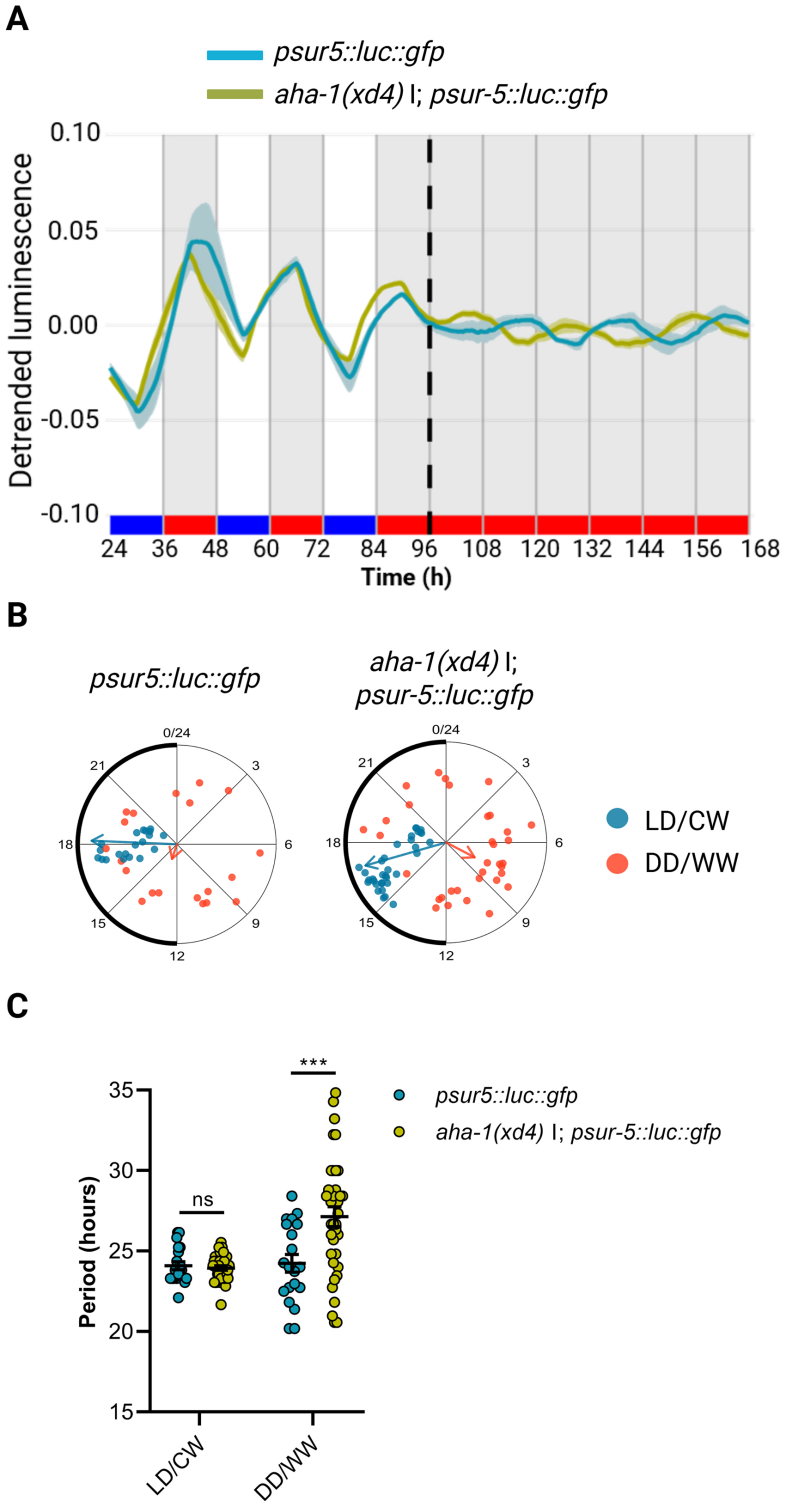
AHA-1 modulates circadian entrainment in adult nematodes. **(A)** Average reporter activity of adult populations under dual cyclic conditions (LD/CW, 15.5/17°C) and FR conditions (DD/WW, 17°C): *psur-5::luc::gfp* (control) and *aha-1(xd4)* I; *psur-5::luc::gfp* mutants. Luminescence signals are shown as mean ± SEM. The average reported activity was displayed with rhythmic populations. Each population consisted of 50 adult nematodes per well. **(B)** Rayleigh plots showing the phase of the bioluminescent peak under dual cyclic conditions (LD/CW, light blue dots) and the first bioluminescent peak on the first day of release to FR (DD/WW, pink dots) for rhythmic population nematodes: control (LD/CW: 18.16 ± 0.30 h, R = 0.95 and DD/WW: 13.23 ± 1.87 h, R = 0.17; *n* = 20) and *aha-1(xd4)* (LD/CW: 16.93 ± 0.38 h, R = 0.92 and DD/WW: 7.87 ± 1.44 h, R = 0.35; *n* = 36). **(C)** Average period of rhythmic populations for control (LD/CW: 24.09 ± 0.25 h; DD/WW: 24.24 ± 0.54 h; *n* = 20) and *aha-1(xd4)* mutant (LD/CW: 23.97 ± 0.14 h; DD/WW: 27.14 ± 0.61 h; *n* = 36). Normality of paired differences was confirmed for both strains (Supplementary Table S1). Sidak’s multiple comparisons test (***p < 0.001).

To examine whether the AHA-1 protein is a component of the nematode molecular clock, we used the luciferase reporter *aha-1::luc::gfp::pest* as a clock output, integrating the construct into the nematode genome by UV radiation (control strain VQ1310 *qvls8*, Supplementary Table S1). We then introduced the reporter into *aha-1* mutant backgrounds by genetic crosses with this control strain (strain VQ1722 *aha-1(xd4)* I; *qvIs8* [*psur-5::luc::gfp + punc-122::RFP*], Supplementary Table S1). Animals were entrained to a dual LD/CW cycle (18.5/20°C), and the bioluminescent activity of the *sur-5* promoter was measured thereafter in adult nematodes over three days in LD/CW (15.5/17°C) cycles and then for three days under constant conditions (DD/WW, 17°C). The control and the *aha-1(xd4)* strains exhibited a *sur-5* promoter rhythm under cyclical and FR conditions, thereby providing evidence that nematodes can be synchronized through a dual cycle of light and temperature (Figure 3A; Supplementary Figure S3). Under FR conditions, *aha-1(xd4)* nematodes showed a peak of luminescence during the subjective day, in antiphase to the control strain (Figure 3A). Both strains exhibited weak entrainment following this protocol, as evidenced by the acrophase shift in LD/CW and DD/WW conditions, suggesting that a masking mechanism is involved (Figure 3B). Although there was considerable high variability of circadian period across the rhythmic mutant populations, we found that *aha-1(xd4)* animals showed a significantly longer period than the control strain (27.14 ± 0.61 h, *n* = 36 mutant rhythmic, *n* total = 71, ~ 51% rhythmic vs. 24.24 ± 0.54 h, *n* = 20 control rhythmic, *n* total = 45, ~ 44% rhythmic) (Unpaired t-test, *p = 0,0256) (Figure 3C). These results suggest that *aha-1* could modulate the period of *sur-5*-driven luminescent rhythms. To strengthen this finding, it would be necessary to evaluate whether a transgene of *aha-1* can rescue the aberrant behavior observed in the *aha-1(xd4)* mutant when overexpressed from an extrachromosomal array under its native promoter.

## 4 Discussion

CLOCK, BMAL1, and CYCLE, which encode gene expression activators, regulate circadian rhythms in metazoans (Patke et al., 2020; Gao et al., 2024). The AHA-1 protein from *C. elegans* possesses all conserved domains and shows high homology with BMAL1 in mammals and CYCLE in *Drosophila*. We examined whether AHA-1 also regulates *C. elegans*’ circadian rhythms by measuring bioluminescence of the rhythmic *sur-5* reporter gene (Goya et al., 2016). Using this tool, we previously showed that fundamental properties of circadian rhythms such as entrainment, rhythmicity under FR, entrainment after a 6-h phase-shift, and temperature compensation, also apply to nematodes (Goya et al., 2016).

Here we found that expression from the *aha-1* promoter is rhythmic with a period close to 24 h, peaking in the middle of the nocturnal phase. We have also tested VQ1324 transgenic animals carrying reporter transgene *plin-42::luc::gfp::pest*. We found that the luminescence rhythms for the *lin-42* promoter were in antiphase to the bioluminescence rhythm of the *aha-1* promoter under cyclic conditions. It is noteworthy that these results coincide with those found in other organisms, such as in mice, where it was observed that the transcriptional rhythms of *per* and *bmal-1* are expressed in antiphase (Takahashi, 2017). Likewise, we did not detect a significant circadian transcription in *aha-1* mRNA during adulthood under our dual light and temperature entrainment conditions. The *aha-1* promoter reporter, maintained as an extrachromosomal array, likely causes overexpression and stronger rhythmic luciferase signals compared to endogenous *aha-1* mRNA levels, which are lower and tightly regulated. As described in the Methods section, the luminescence assay was performed in liquid medium without agitation, whereas the qPCR experiment was carried out in liquid medium with agitation. Differences in experimental conditions—such as medium agitation and oxygen availability—may explain the observed shift in peak timing between promoter-driven activity and endogenous transcript expression, given AHA-1’s role in hypoxia response (Takahashi, 2015). Previous studies have shown that *aha-1* mRNA transcriptional levels are not rhythmic in the adult nematode, at least under classical light–dark or temperature cycles (van der Linden et al., 2010; Jiang et al., 2001). In *C. elegans*, *period* and *casein kinase 1ε/δ* are conserved as *lin-42* and *kin-20*, respectively. Our lab and others have shown that these genes do not exhibit circadian rhythmicity at the mRNA level, at least in total RNA extracts of whole adult animals (Lamberti et al., 2024; van der Linden et al., 2010; Jiang et al., 2001; Hiroki and Yoshitane, 2024). Finally, it remains possible that orthologous clock genes do cycle in a small subset of cells or tissues in worms and that this get lost from bulk RNA extraction. Follow up experiments using scRNA-Seq (single-cell RNAsequencing) or tissue-specific RNA-seq would be needed.

*bmal-1* knockout mutant mice exhibit a decrease or lack of behavioral circadian rhythmicity, as well as a reduced lifespan (Olmedo et al., 2012; Kondratov et al., 2006). In *Drosophila*, *cycle* mutants show disruptions in the sleep period and also a shortened lifespan in males (McDearmon et al., 2006). The involvement of *bmal-1* in lifespan appears to be conserved among animals, since *aha-1* mutants have been shown to exhibit a decreased lifespan too (Olmedo et al., 2012). In *C. elegans*, null mutations for *aha-1* are lethal (Hendricks et al., 2003). In this work we have demonstrated that a decrease in the expression levels of *aha-1* lengthens the period of transcriptional rhythms at the *sur-5* promoter in *aha-1(xd4)* mutant; however, the ability to synchronize with the zeitgeber was comparable to the control strain, as they displayed a similar dispersion of acrophases in LD/CW and DD/WW conditions. Therefore, our data demonstrate that *aha-1* is necessary for determining the period of circadian rhythms in *C. elegans* but its role in circadian entrainment remains to be fully determined.

We have also studied the role of *lin-42* (homolog to the *per* gene) in the circadian rhythms of the adult nematode by recording locomotor activity and measure the luminescence (Simonetta et al., 2009; Lamberti et al., 2024). Locomotor activity recording (Qin et al., 2006) and the bioluminescence reporter (Goya et al., 2016) assays, both developed by our laboratory, allow us to advance in understanding the molecular basis of *C. elegans*’ circadian clock. However, the bioluminescence reporter proved to be more reproducible than locomotion-based methods, allowing to record robust circadian rhythms in gene expression that persist even after 2 days in FR conditions (Migliori et al., 2023; Goya et al., 2016). The circadian rhythms of locomotion do not appear to be robust since they do not persist for more than 1 day under FR conditions. One of the possible reasons for this could be that the signal-to-noise ratio is low and makes it difficult to record for long periods of time.

In summary, we show that the CYCLE/BMAL-1 homolog AHA-1 plays a key role in the generation of circadian rhythms in *C. elegans* adult nematodes, mainly by regulating period length. Based on results from our laboratory (Lamberti et al., 2024), AHA-1 could act as a transcriptional activator of LIN-42, as is the case for its homologs BMAL-1 and PER in mammals and *Drosophila* (Simonetta and Golombek, 2007; Partch et al., 2014). These results suggest that the molecular control of circadian regulation in *C. elegans* exhibits some similarities to other clock model systems.

## Data Availability

The raw data supporting the conclusions of this article will be made available by the authors, without undue reservation.

## Footnotes

^1^ https://ispiousas.shinyapps.io/circaluc/

^2^ https://app.biorender.com/

^3^ http://mengwanglab.org/atlas
