# Supplementary material for: Role of the clock gene homolog aha-1 in the circadian system of Caenorhabditis elegans
Source: Front Neurosci. 2025 Jun 24;19:1618370. doi: 10.3389/fnins.2025.1618370 (PMC12235174; doi:10.3389/fnins.2025.1618370)
Supplement: Supplementary file 1 [file Data_Sheet_1.DOCX]

Supplementary Material


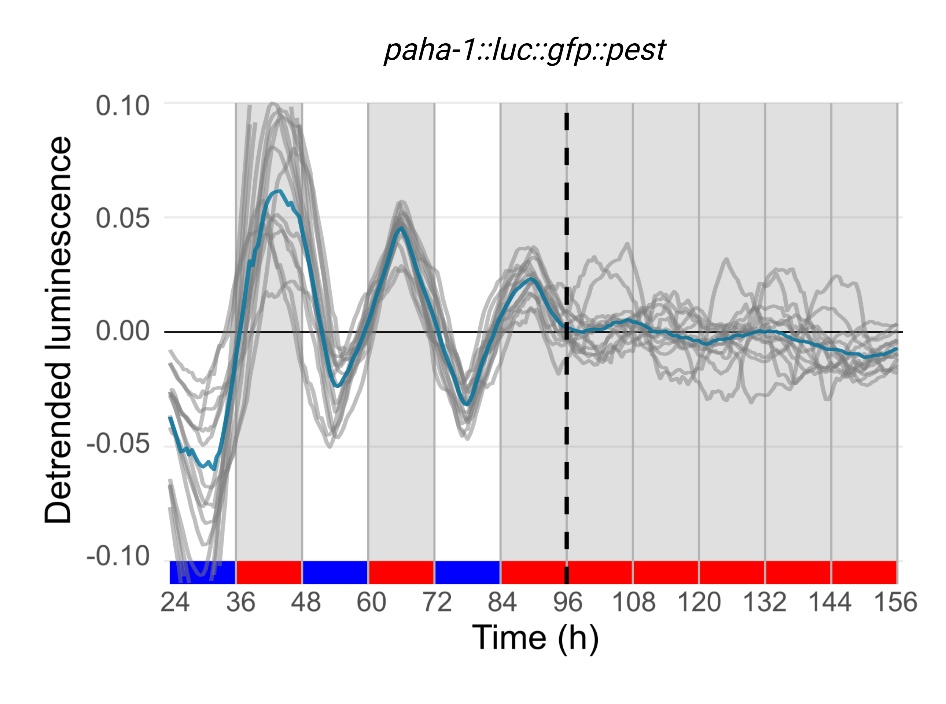


**Supplementary Figure 1.** Reporter activity of all adult populations under dual cyclic conditions (LD/CW, 15.5/17 °C) and FR conditions (DD/WW, 17 °C). Luminescence signals are shown as mean ± SEM in blue line and all the individual wells are represented in the gray line. VQ1071 *qvEx295* [*paha-1::luc::gfp::pest*] strain, n total = 30. Each population consisted of 50 adult nematodes per well.


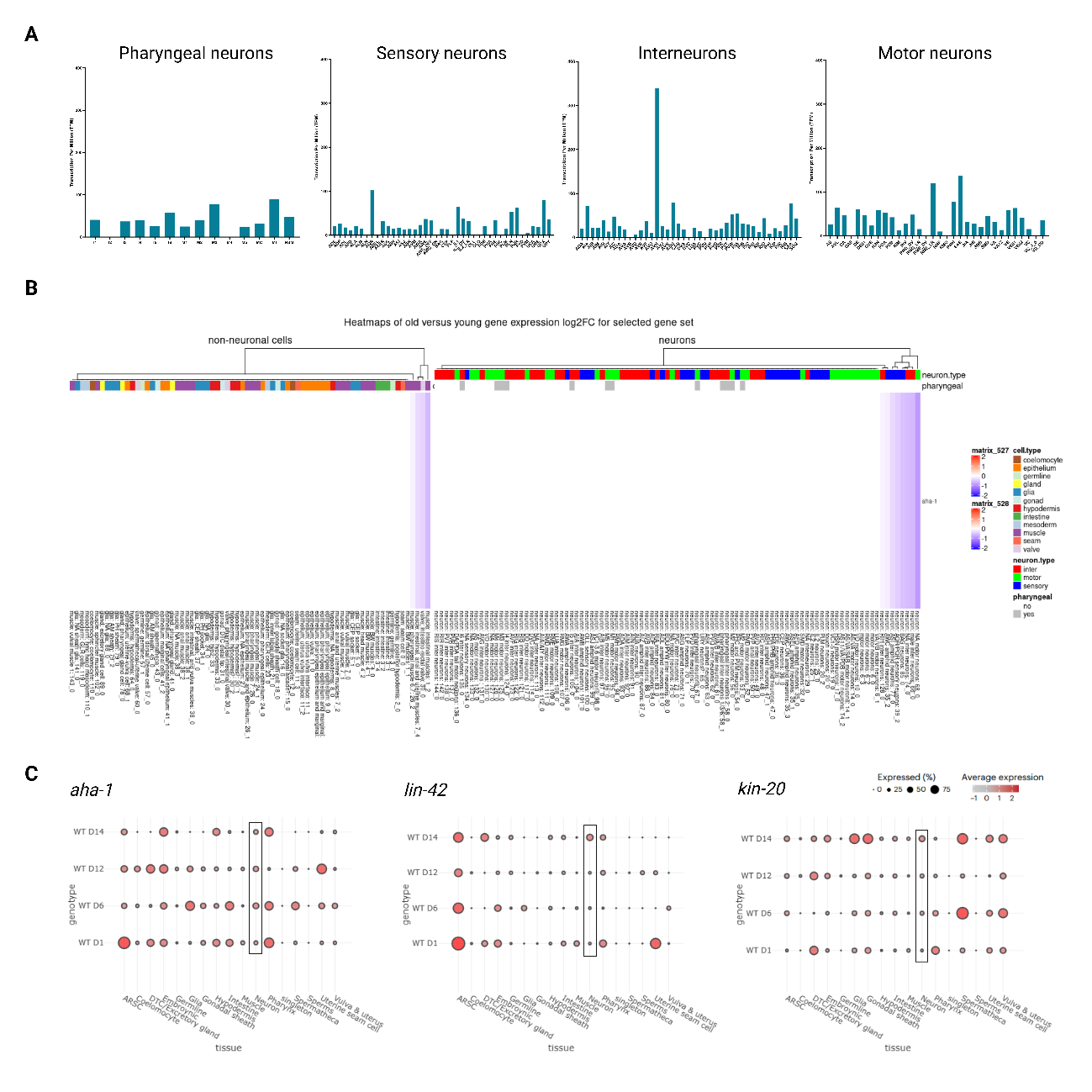


**Supplementary Figure 2.** Expression of *aha-1* by single-cell RNA-seq. **(A)** Representative expression of *aha-1* gene in sensory neurons, interneurons, motor neurons, and pharyngeal neurons. Data obtained from CeNGEN (<https://www.cengen.org/>). The scale represents the percentage of expression of the genes of interest. TPM stands for transcription per million. Each bar represents a neuron. **(B)** Heatmap of gene expression changes (log2FC between days 8,11,15 and days 1,3,5) across all cell type clusters for *aha-1* gene. The heatmap was from obtained (<https://c.elegans.aging.atlas.research.calicolabs.com/embeddings>). **(C)** Dot plot visualizing the cell-type-specific expression patterns of the *aha-1*, *lin-42*, and *kin-20* genes, based on publicly available RNA-seq data from the *C. elegans* Aging Atlas (<http://mengwanglab.org/atlas>). Dot size represents the percentage of cells within a specific tissue expressing the gene, and the color intensity indicates the average expression level of the gene across the tissue. D: day; WT: wild-type strain.


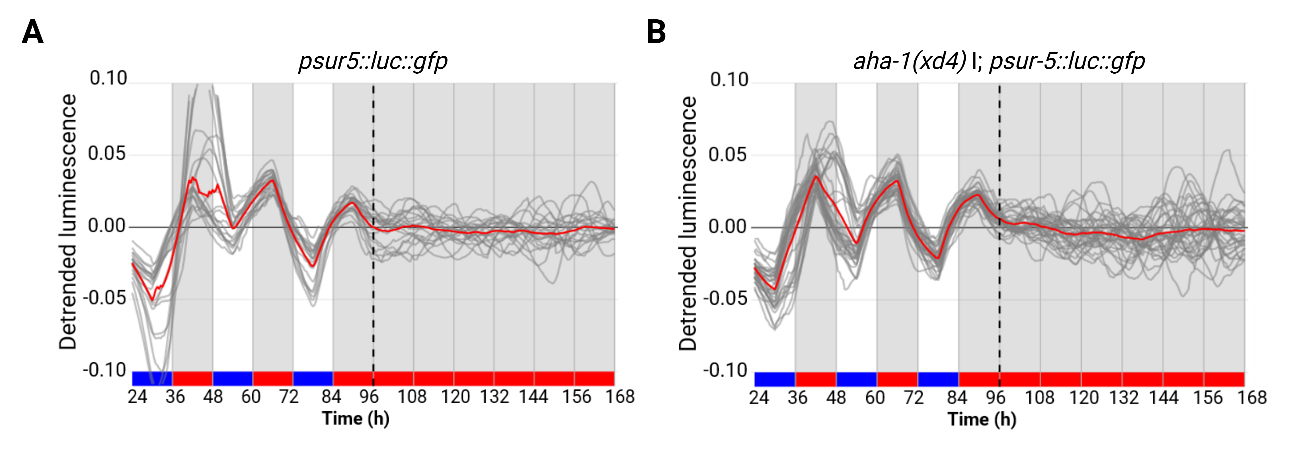


**Supplementary Figure 3.** Reporter activity of all adult populations under dual cyclic conditions (LD/CW, 15.5/17 °C) and FR conditions (DD/WW, 17 °C). Luminescence signals are shown as mean ± SEM in red line and all the individual wells are represented in the gray line **(A)** control, n total = 39, **(B)** *aha-1(xd4)*, n total = 72. Each population consisted of 50 adult nematodes per well.
