# Supplementary material for: Role of the clock gene homolog aha-1 in the circadian system of Caenorhabditis elegans
Source: Front Neurosci. 2025 Jun 24;19:1618370. doi: 10.3389/fnins.2025.1618370 (PMC12235174; doi:10.3389/fnins.2025.1618370)
Supplement: Supplementary file 2 [file Table_1.DOCX]

| **Strain** | **Genotype** | **Mean**  **period* ± SEM**  **(LD/CW)** | **Mean**  **period* ± SEM**  **(FR)** | **Shapiro-Wilk p**  **(LD/CW−DD/WW)** | **N**  **Total** | **N**  **Rhythmic** |
| --- | --- | --- | --- | --- | --- | --- |
| VQ1071 | *qvEx295* [*paha-1::luc::gfp::pest*] | 23.76 ± 0.25 h | 24.88 ± 1.00 h | 0,3940 | 30 | 14 |
| VQ1324 | *qvEx361* [*plin-42::luc::gfp::pest*,  *pCFJ90*] | 24.55 ± 0.97 h | 24.59 ± 1.14 h | 0,2678 | 26 | 9 |
| VQ1310 | *qvIs8* [*psur5::luc::gfp + punc-122::RFP*] | 24.09 ± 0.25 h | 24.24 ± 0.54 h | 0,2732 | 45 | 20 |
| VQ1722 | *aha-1(xd4)* I; *qvIs8* [*psur-5::luc::gfp* + *punc-122::RFP*] | 23.97 ± 0.14 h | 27.14 ± 0.61 h | 0,2586 | 71 | 36 |

**Supplementary Table 1.** Summary of entrainment conditions, sample sizes, and statistical data. Number of samples (N Rhythmic = numbers of samples exhibiting circadian rhythmicity under free-running conditions, N Total = total number of samples; 1 sample = 1 well of a 96-well plate), and strains. * Period was calculated over the rhythmic populations as described in Material and Methods. LD/CW: 12:12 h (15.5/17 °C). FR (free-running): 17 ºC, DD. Shapiro-Wilk test, p > 0.05 (normally distributed).

| **Analysis Method** | **Strains and Mean period* ± SEM** | | | |
| --- | --- | --- | --- | --- |
|  | **VQ1310** | **VQ1722** | **VQ1071** | **VQ1324** |
|  | **LD/CW 12:12 h (15.5/17 °C) conditions** | | | |
| LS – Lomb-Scargle | 25.54 ± 1.17 h | 23.97 ± 0.42 h | 24.53 ± 0.51 h | 26.67 ± 1.82 h |
| MESA – Maximum Entropy Spectral Analysis | 22.84 ± 0.27 h | 23.11 ± 0.19 h | 23.15 ± 0.39 h | 24.56 ± 1.29 h |
| MFourFit – Multiple Fourier Fit | 25.52 ± 1.11 h | 24.00 ± 0.40 h | 24.40 ± 0.49 h | 26.67 ± 1.77 h |
|  | **DD/WW (17 °C) conditions** | | | |
| LS – Lomb-Scargle | 24.15 ± 0.57 h | 27.08 ± 0.64 h | 24.46 ± 1.07 h | 22.61 ± 0.94 h |
| MESA – Maximum Entropy Spectral Analysis | 23.44 ± 0.59 h | 26.20 ± 0.68 h | 23.43 ± 0.74 h | 22.52 ± 2.06 h |
| MFourFit – Multiple Fourier Fit | 25.19 ± 0.81 h | 27.46 ± 0.70 h | 27.55 ± 1.72 h | 26.90 ± 1.98 h |

**Supplementary Table 2.** Period estimation by different algorithms. All analyses were performed using the BioDare2 platform (<https://biodare2.ed.ac.uk/>). *Period was calculated over the rhythmic populations (VQ1310, n = 20; VQ1722, n = 36; VQ1071, n = 14 and VQ1324, n = 9). Normality (Shapiro-Wilk test) was not met across period estimates; therefore, a non-parametric test (Kruskal-Wallis test) was applied. No significant differences were found between periods obtained with different periodogram methods.
